# What it takes to be a “Good” correctional officer: Occupational fitness and co-worker expectations from the perspective of correctional officer recruits in Canada

**DOI:** 10.1177/17488958221087488

**Published:** 2022-04-11

**Authors:** Marcella Siqueira Cassiano, Brittany Ann Bennett, Elizabeth Andres, Rosemary Ricciardelli

**Affiliations:** Memorial University of Newfoundland, Canada

**Keywords:** Accountability, confidence, correctional work, co-work expectation, job role, occupational fitness, reliability

## Abstract

Selecting individuals who are the right “fit” for correctional work is not an easy task for prison administrators because of the dangerous nature of correctional work and the centrality of prison employees in the prisoner’s rehabilitation process. We analyze fitness for correctional work from the employee’s perspective, complementing the scholarship focused on the employer’s view. We measure occupational fitness in terms of co-worker expectations, analyzing 104 semi-structured interviews conducted with Federal Canadian Correctional Officer recruits in 2018/2019. Recruits in our sample expected a correctional officer to be accountable, reliable, and confident. Understanding the mind-set of new hires provides insights into the correctional officer role and allows employers to align employer-employee expectations, as well as review training and recruitment, which can improve the employee well-being and reduce turnover rates.

## Introduction

Correctional work entails performing a role that many people would find challenging and dangerous. The “role” refers to the duties and rights that are attached to any status (Goffman, 1959: 34) including occupational statuses. In exchange for payment, limited recognition, and the “implicit suggestion” that caring for prisoners might be rewarding in itself (Liebling et al., 2011). Correctional officers (CO) work long shifts in confined spaces and regularly deal with risks that can affect their mental and physical health and safety. The risks that COs experience when fulfilling their responsibility pertaining to the “care,” “custody,” and “control” of prisoners (Hogan and Lambert, 2020) include the possibility of being victimized (e.g. assaulted) (Konda et al., 2012;, Ricciardelli 2019a) and exposed to potentially psychological traumatic events. Such risks are persistent, which become a reality at some point in the COs’ careers, can result in officers’ burning out and developing mental health injuries (Armstrong and Griffin, 2004; Bierie, 2012; Carleton et al., 2019). Particularly, COs become vulnerable when responding to overdoses, altercations, fecal bombing, stabbings, self-harming, and deaths by suicide (Armstrong and Griffin, 2004; Carleton et al., 2019; Mitchell et al., 2000;, Ricciardelli 2019a; Ricciardelli and Power, 2020). Understanding the CO role has particular importance to prison researchers because the success of the criminal justice system in rehabilitating criminalized persons and preventing recidivism depends, although not exclusively, on how COs perceive and perform their occupational duties. In this study, we inquire into how COs interpret and accomplish fitness to correctional work, that is, the officer’s suitability to perform the correctional role.

Scholarly concern for the CO role emerged in the late 1960s, when prisoner rehabilitation became ideal within correctional services in the US and a core component of correctional work (Carroll, 1974; Crouch, 1980; Frank, 1966; Toch, 1978). Initially, prison researchers, especially those who are working for correctional institutions in the US, explored the CO’s ability to participate in the prisoner’s resocialization by multitasking and acting both as a custodial (i.e. “prison keeper” or “screw”) and a treatment specialist (Frank, 1966). Once that ability was established and correctional institutions started engaging prison guards in the role of correctional agents, scholars started analyzing and defining the new officer role components (Crawley and Crawley, 2008; Crawley, 2004; Toch, 1978). Researchers anticipated that the personal characteristics and role difficulties (e.g. conflict, strain, overload, and stress) (Philliber, 1987) would impact the officers’ views of the prison and prisoners, ultimately affecting their ability to build relationship (i.e. rapport) with prisoners (Toch, 1978) and promote rehabilitation.

The elevation of officers to the role of “enablers” of rehabilitation led to the development of numerous lines of inquiry within the field of prison studies, many of which are still active. These inquiries produce a significant knowledge of the topics that underpin correctional work, from recruitment (Morgan and Smith, 2009; Stickrath and Sheppard Jr, 2004) and retention (Jurik and Winn, 1987; Lambert et al., 2006; Mitchell et al., 2000; Slate et al., 2001; Sproule and Berkley, 2001; Wright, 1994) to professional orientation (Cullen et al., 1989; Farkas, 1999; Ferdik, 2017; Whitehead et al., 1987) and job satisfaction (including dissatisfaction in the form of stress and/or burnout) (Armstrong et al., 2015; Cullen et al., 1985; Lambert et al., 2002). The field of prison studies has been marked by the continual revision and reassessment of the CO role (Hogan and Lambert, 2020; Liebling et al., 2011), as well as discussions on how this role is transmitted among officers through the prison staff sub-cultures (Arnold et al., 2007; Farkas and Manning, 1997; Klofas, 1984). A variety of metrics designed to measure the officer’s attitude toward prisoners, correctional work, and prisons (Tellier et al., 2001) are emerged, allowing researchers to quantify how COs exercise their job roles.

Recruitment and screening metrics have also emerged to help correctional managers to assess the attributes that has become valued in a recruitment, such as interpersonal sensitivity, higher intelligence, self-insight, and flexibility in the enforcement of rules and procedures (Liebling and Arnold, 2004; Lough et al., 2007). Nonetheless, the literature dedicated to correctional work fitness was limited and focused on the correctional employer’s perspective and interpretation of the CO role (Morgan and Smith, 2009; Shusman and Inwald, 1991; Sproule and Berkley, 2001; Stickrath and Sheppard Jr, 2004).

The desire to understand the CO role from a comprehensive perspective and helps in guaranteeing the officer’s ability to uphold constructive interactions with prisoners led to several researchers in the US (Toch, 1978), the UK (Arnold et al., 2007; Liebling et al., 2011), and Denmark (as cited in Arnold et al., 2007; Liebling et al., 2011) to inquire into the existence of a “role model” officer. In search of an “ideal fit” for correctional work, prison researchers adapted (Gilbert, 1997) or applied (Arnold et al., 2007; Liebling et al., 2011) descriptive typologies that summarize the officer’s personality, skills, and decision-making process (e.g. “professional,” “reciprocator,” “enforcer,” and “avoider,” see (Gilbert, 1997; Muir, 1977) often finding that no single, unifying characteristic defines a “good officer.” This finding revealed that COs form a diverse, as opposed to a “monolithic,” workforce (Arnold et al., 2007; Gilbert, 1997; Liebling et al., 2011; Philliber, 1987; Toch, 1978). Officers considered as “good” by co-workers and prisoners presents a diverse range of attributes such as confidence, integrity, honesty, empathy, compassion, caring, reflexivity, good judgment, and flexibility (Arnold et al., 2007; Liebling, 1999; Liebling et al., 2011; Toch, 1978). Regardless of their findings, studies on fitness to correctional work (Arnold et al., 2007; Liebling, 1999; Liebling et al., 2011; Toch, 1978) center their analysis in trying to understand the staff-prisoner relationship, which is the “heart” of prison work (Liebling et al., 2011). They aim to identify the factors that influence the officer’s ability to develop a good relationship with prisoners, such as the officer’s “personality” or “skills” (Liebling et al., 2011) — to help make the correctional work more conducive to rehabilitation.

We aim to understand the role of the CO, like our peers in prison research. We recognize that understanding the occupational fitness in direct association with the management of prisoners is a pathway to create knowledge that can help improve the experience and effectiveness of incarceration. However, we take a distinctive, albeit complementary, analytical pathway. Based on the assumption that the staff-prisoner relationships and prison work conditions reflect each other (Liebling and Arnold, 2004; Ricciardelli and Power, 2020), we draw on tradition of inquiring into the “role model” officer and the attributes outlining a “good fit” to explore how COs are related to prison work (i.e. how they view their job and the expectations they have) and their work conditions. Our motivation is not finding a role model personality within correctional work, instead, we use inquiries into occupational fitness as a methodological strategy to capture peer expectations and thus gain a better understanding on how COs’ perceive, experience, and perform their role.

Specifically, our line of inquiry intends to understand how COs perform their role despite the challenges that they face. We also aim at identifying constitutive elements of the CO role that can hinder or facilitate the officer’s ability to perform prison work. Inquiring among officers about the qualities that form a “good officer” can provide a window into how correctional work is evolving, including the responsibilities, risks, and vulnerabilities that mark correctional work and how individuals of different demographic groups perform correctional work. The knowledge of correctional officer recruits (CORs) expectations can also apply in understanding the role of training in the transmission of the CO role and prison staff cultures (Duffee, 1974; Ingel, 2020; Klofas and Toch, 1982; Klofas, 1984; Owen, 1984) within correctional services. Role inquiries based on views and perceptions can also help to improve recruitment practices, which includes aligning the CORs’ expectations with the realities of the job and assessing whether the training has satisfied the needs of correctional services with regards to the understanding of the qualities that are necessary to become a good officer.

Our occupational fitness analysis is based on 104 interviews with CORs in Canada, that is the officers in charge of prisoners those are sentenced to two years or more in custody. Interviewing recruits on the CO role gives researchers a vantage point to understand the contents of the CO role and capture the role at its germinal state, which allows for a deeper understanding of how it evolves. CORs enter occupational positions with expectations that is clearly manifested in their perceptions of how individuals, including themselves and their co-workers should think, behave, and perform at work. For the most part, such expectations result from a process of enculturation that happens during training and recruitment (Arnold et al., 2007). For individuals being recruited to Canada’s federal prisons, this process comprises a 14 week training program referred to as Correctional Training Program (CTP), organized and provided by Correctional Services Canada (CSC). Participating in CTP entails being immersed in job training at the academy, living and learning with their cohorts of recruits, as well as learning the CO role.

## The literature on occupational fitness and peer-expectation

Often situated in management, human resources, and labor sociology, the broad literature on occupational fitness and peer expectations views self and co-worker perception in the workplace as a factor that can hinder or facilitate labor productivity, as opposed to a signifier occupational role and work conditions. For instance, the literature views in co-worker perception of ethics and leadership (Morgan, 1993), performance (Hwa, 2012; Williams and Karau, 1991), trust (Chung and Jackson, 2011; Lehmann-Willenbrock et al., 2012; Worley et al., 2018), and support (Bateman, 2009; Bradley et al., 2019; Chung and Jackson, 2011; Mitchell et al., 2000; Worley et al., 2018) as a catalyst for knowledge sharing, teamwork, innovation, commitment, and work-life balance. Further, the literature on peer expectation has paid particular attention to peer support and trust as correlates of job satisfaction (Bateman, 2009; McCalister et al., 2006) and productivity (Bradley et al., 2019; Chung and Jackson, 2011; Ferres et al., 2004; Williams and Karau, 1991).

Based on the literatures available, peer support and co-worker trust predict employee perceived organizational support, turnover intention, as well as employee’s effective commitment to the job, including team and organizational commitment (Ferres et al., 2004; Lehmann-Willenbrock et al., 2012). Co-worker trust, which is intertwined with peer support, also represents a significant pathway connecting the employee appreciation of age diversity to employee well-being (i.e. stress and work-life balance) and team commitment (Lehmann-Willenbrock et al., 2012). Furthermore, the trust among co-workers can enhance the creativity and collaborative work (Chung and Jackson, 2011). Co-worker support was a significant predictor of workplace stress in high-performance workplaces (e.g. tech companies) and government jobs (McCalister et al., 2006). Despite the significance of support and trust across co-worker expectation literature more broadly, less attention has been directed toward understanding the function of trust in high-risk work contexts (Conchie et al., 2006), like in the prison environment. Meanwhile, negative co-worker expectations has been associated with several factors, including compromised organizational citizenship behavior (Turnley and Feldman, 2000), emotional exhaustion (Wang et al., 2018), workplace stress (McCalister et al., 2006), and turnover intentions (Bateman, 2009; Ferres et al., 2004).

Unmet expectations, like negative expectations can also create workplace problems. According to Porter and Steers (1973), when employees encounter something other than what they expected of their occupational position, they have encountered *unmet expectations*. There is evidence that unmet job expectations increases psychological and physical health concerns (Maden et al., 2016; Proost et al., 2012; Taris et al., 2006), job stress (Lait and Wallace, 2002), turnover intentions (Maden et al., 2016; Orrick, 2008; Proost et al., 2012; Taris et al., 2006; Turnley and Feldman, 2000), and employee organizational commitment and culture (Orrick, 2008; Turnley and Feldman, 2000).

Within the world of correctional work and beyond the scholarship as cited in the introduction, studies that aim at understanding occupational fitness from the perspective of peer expectation are rare. Scholars in correctional studies often mention peer expectation in their analysis of correctional work experience and performance, but peer expectation appears “disguised” under its concrete outcome, such as peer support and management support. In numerous studies, peer support, alongside age, gender, ethnicity, education, and experience, is considered as a determinant of job satisfaction, job stress, and organizational commitment (Butler et al., 2019). A few studies in the US have shown that the peer support predicts turnover intentions among workers in juvenile correctional facilities (Mitchell et al., 2000). Co-worker and management action and inertia have emerged as a significant, yet little explored theme related to perception of occupational risk during interviews with provincial and territorial COs in Canada, (Ricciardelli2019a). Across the CO literature, researchers have identified the positive effects of co-worker support and trust on the overall workplace environment and “collective efficacy” (Worley et al., 2018), meaning informal social control mechanisms based on mutual trust and collective expectation (Shaw and McKay, 1942). Especially, the association of peer support and trust is relevant for COs. In the case of correctional work, an inherently dangerous and stressful occupation, not being able to trust and rely on co-workers can become a significant source of stress and an occupational safety concern (Ferres et al., 2004;, Ricciardelli2019a); trust and reliability in this line of work is conducive to feel safe on job.

Our study complements the field of correctional work by analyzing peer expectation as a mean to understand the CO role and by approaching occupational fitness from the employee’s perspective. In following sections, we present the methods that we used to analyze this article, our findings, and discussion, which includes our conclusions.

## Methods

The data supporting our analysis derives from a multi-year, mixed-method study (2018–2028) on the mental health and well-being of COs in Canada’s Federal Prison System. A longitudinal study collects both qualitative (interviews) and quantitative (surveys) data on officers when they are recruited (i.e. baseline interviews) and annually (i.e. follow-up waves). The 104 interviews used to support this article were collected at the National Training Academy of the Correctional Service Canada in Kingston, Ontario, between August 2018 and March 2020.

Following a semi-grounded conversational format (Charmaz, 2014; Glaser and Strauss, 1967), the interviews asked about various topics related to correctional work to provide insights into the high prevalence of operational stress injuries (Oliphant, 2016) among the COs. Such topics included, among other themes, the officers’ occupational expectations, experiences, perceptions, concerns (e.g. stressors and challenges), exposure to potentially psychologically traumatic events, as well as their views of prison, prisoners, and co-workers. Views of co-workers included the question that originated the excerpts analyzed in this article: “what are the qualities in a correctional officer that you feel would be most respected by other COs?” Early in the study, this question was phrased using CTP as context: “When you look around at the people in your CTP, are there people that will make really good officer? Why?” Inquiring into peer perception, the question was designed to capture the recruit’s expectations and understanding of the CO role and provide insights into how CORs experience correctional work.

CSC facilitated the participant recruitment and data collection by advertising our project to CO recruits and provided a private space for the project team to conduct interviews. The interviews, which lasted between 45 and 90 minutes, were voice recorded and transcribed verbatim. All participant identifying information was anonymized and their names were replaced with a participant identification number. Despite CSC’s collaboration, participation in our project was voluntary. Also, CSC had no access to the primary research data whatsoever. The research’s ethics protocols has received approval from the Memorial University of Newfoundland (File No. 20190481).

Applying a consistent process of data coding is crucial to an accurate interpretation. All interviews were coded in a three-step process. First, we used the software NVivo to axial-code (Kendall, 1999) the interviews according to a multi-item coding scheme that reflected the themes explored in the interviews. This scheme included a category labeled “occupational fitness” and a sub-category labeled “qualities in an officer,” under which we coded the answers analyzed in this article. Second, we used open-coding (Cascio et al., 2019) to analyze the answers under “qualities in an officer,” looking for patterns and repetitions and classified them into themes based on their most emphasized quality. Three themes were emerged in their answers: accountability, reliability, and confidence. We excluded 15 participants from the analysis; their answers were unclear or who were not asked the question, remaining with a total of 104 cases. Finally, we tabulated the participant demographics and the themes emerging from the coding process (i.e. “expected quality in a CO”) into the software IBM-SPSS and examined the data for patterns involving gender, age, and previous correctional experience. Our data set has comprised of 55 males and 49 females. About half of them were aged between 25 and 34. Approximately 40% of recruits (46 individuals) had correctional work experience prior to being recruited into CTP, usually working in Canada’s provincial or territorial correctional system (Table 1). Expecting to find an association between the variable “expected quality in a CO” and the variables “correctional work experience” and “gender,” we calculated the chi-square test of independence for the following variables: quality emphasized by the participant, gender, age, and correctional experience (Table 2).

**Table 1.** Participant characteristics.

	Recruits	Percent
*Sample*	104	100
*Gender*		
Male	55	52.9
Female	49	47.2
*Age Group*		
19–24	26	25.0
25–34	51	49.0
35–44	18	17.3
45–54	8	7.7
45–64	1	1.0
*Correctional Experience*		
Yes	46	38.7
No	73	61.3
*Occupational fitness (perception of respected qualities by COs)*		
Accountability	53	51.0
Reliability	35	33.7
Confidence	16	15.4

**Table 2.** Expected quality in a CO in association with gender, age, and correctional experience.

	Accountability	Reliability	Confidence	Chi-square results (at 0.95)
*Gender*				
Male	32 (60.4)	18 (51.4)	5 (31.2)	X^2^ (2, N = 104) = 4.2, p = .121
Female	21 (39.6)	17 (48.6)	11 (68.8)	
*Age Group*				
19–24	8 (15.1)	11 (31.4)	7 (43.8)	X^2^ (8, N = 104) = 14.0, p = .080
25–34	27 (50.9)	16 (45.7)	8 (50.0)	
35–44	14 (26.4)	3 (8.6)	1 (6.3)	
45–54	4 (7.5)	4 (11.4)	-	
45–64	-	1 (2.9)	-	
*Correctional Experience*				
Yes	19 (35.8)	12 (34.3)	9 (56.3)	X^2^ (2, N = 104) = 2.5, p = .279
No	34 (64.2)	23 (65.7)	7 (43.8)	

## Findings

Most participants (51.0% or 53 individuals) named accountability as the most respected quality, while reliability and confidence were stressed by 33.7% (35 individuals) and 15.4% (16 individuals), respectively (Table 1). Overall, the participants expected “good officers” to do their job, do it right, own their actions, show some confidence, and have their co-worker’s back. We found no significant quantitative (measured in chi-squares) or qualitative (visible patterns in answers) dependence between the perception of fitness to correctional work and gender, age, and correctional experience within the sample. In this section, we discuss three themes that are emerged in the interviews and explore their meanings.

### Accountability

Accountability represents a foundational element in organizations. Among other accomplishments, the culture of accountability generates compliance to workplace rules and protocols (Han and Perry, 2020). Accountability is grounded in the premise that employees monitor and evaluate each other’s behaviors against objective institutional rules and codes of conduct, rewarding desirable behaviors and sanctioning undesirable ones (Erichsen and Reynolds, 2020; Han and Perry, 2020). However, the practice of accountability also includes a subjective element that is still understudied (Han and Perry, 2020). Such an element refers to the employee interpretation and internalization of accountability systems, which vary among individuals.

Participants discussed accountability from three empirically different, but intertwined perspectives, which are referred in the literature as “performative,” “attributability,” and “answerability” (Han and Perry, 2020). The performative perspective refers to executing an action (“doing your job”). Attributability links the performed action to an actor (“owning your mistakes”). At the same time, answerability expects the actor to justify the decision taken in their job according to protocols (“doing the job right”). Participants clearly illustrated at least one of these dimensions when describing accountability as a commendable attribute in COs.

#### Performance

Emphasizing the performative side of accountability, numerous participants reported that getting the job done would be the primary “quality” the officers respect in their peers. For example, P93 represented such quality as “not leaving the work for other people,” that is, performing a task to its entire extent. She drew on previous work experience to highlight that people do not need to go “above and beyond,” but they need to complete the tasks under their responsibility: “It’s not like you have to go extra mile or anything. . . if you finish whatever you’re supposed to finish during your shift, it’s very much appreciated, cause I have personal experience with people who just leave their work. . .” Using a similar rationale, P95 said that COs respect peers who demonstrate “willingness to do the job,” while P89 equated to “showing up” and “doing the job appropriately.” Like P95 and P89, P106 believed that officers expected peers to “show up” and “do” their jobs. Complementing his fellow recruits, P90 outlined the consequence of not working properly: “If you haven’t had work all done for somebody who’s coming in after you, they are going to hate you forever.” According to P106, showing up to complete the task meant working as a “team” to ensure that everyone goes home “safe,” an element that appeared more frequently among participants who highlighted “reliability” as the main attribute respected in peers.

A few recruits associated are not showing up, not doing the job, not taking the initiative as “laziness,” establishing a clear connection between the performative side of accountability and hard work. When inquired if a specific colleague in her CTP cohort would “make a good officer,” P119 answered, “no, cause he’s very lazy, it’s kind of like his predominant trait.” Similarly, P45 took hard work for granted, treating it as a noticeable characteristic of accountability: “Well, everybody knows [that] no one likes a lazy partner.” Following up, she defined a “lazy” person as “someone who has never put in the work, and doesn’t take initiative,” someone who completes their shift and their tasks without finding “ways to get out of [their work].” P119 and P45’s words confirm the connection between accountability and hard work.

Some officers conflated accountability, particularly its performative facet with professionalism, respect, integrity, and honesty. P10 portrayed work avoidance to “dicking around” with co-workers, which he viewed as “dishonest.” To emphasize his point, this participant said that officers would not respect co-workers who “goofed around” and avoided “doing their counts and stuff.” P58 replied that “respect is a “big thing,” but she conditioned respect to “getting the job done.”

Emotional labor, that is, managing emotions and life to keep people around content (Hochschild, 1989; Hochschild, 1983), appeared as an enabler or an associate of accountability. The presence of emotional labor within the performative facet of accountability signals the intellectual complexity of correctional work. P10 said that people had to not only do their job but also enjoy it: “You want to do your job and have fun doing it.” P27 reported a “raunchy, messy sense of humor” as a “quality” in the prison environment. P29 praised a classmate in his CTP for being able to “laugh along and be there when things were said that shouldn’t be said,” explaining that people like his classmate would make a “really good” officer. Trying to clarify his view, P29 explained that his colleague was able to “be there without crossing the line,” meaning fitting in and participating in jokes without being unpleasant to others. Likewise, P13 suggested that “maintaining a sense of humor in a place that probably sucks it out of you” opposes a “bigoted mentality” and forges teamwork, illustrating the creative side of emotional labor. P91 said that COs admire peers that do their jobs “and” stay out of the “rumor mills,” which also takes a degree of emotional labor and an awareness of their social environment.

#### Attributability

As indicated in the specialized literature, accountable employees expect their contributions, activities, and errors to be linked to them (Han and Perry, 2020). A concrete illustration of this “linkage” is in P45’s interview when she equated hard work to leave a “paper trail,” that is a link between employees and the work they do: “If you have an incident, write your report. Don’t try to find ways to get out of it. Because if there’s a bigger problem down the line, and you don’t have that paper trail, like, you just—you know what I mean—don’t be lazy.” Participants often portrayed the link that underpins attributability as “owning mistakes,” being “honest” and “trustworthy,” having “integrity,” and being predictable. Several participants expected officers to respect predictable peers and people who “do what you say you’re gonna do” (P77). Drawing on his life experience, P132 said, “the ability to follow through on what you say you’re going to do that’s the biggest one I’ve heard my whole life.” Meanwhile, P55 described accountability as the same idea as “people . . . who can make decisions and stand alongside it.”

A few participants discussed appearance in connection with attributability, demonstrating that being accountable presupposes a link between the working body and the job performed. P112 said: “yeah just again being maybe professional always. . . that is you keep your hair clean, uniform tidy, and hold yourself and present yourself well.” After discussing the self-presentation-occupation link (e.g. tidy uniform, polished boots), P79 suggested that the COs should be freed from their attributability once they leave the workplace. Suggesting a spatial/temporal separation of “life” and “work” that hardly exists today, P79 continues: “They go into work and their [are] really professional and then outside it’s like they can do whatever and have a good time, drink beers, BBQ, like be totally. . . [do things that] maybe [is] not what you would expect of like someone like that….” P79 also equated accountability and professionalism to “taking pride in your job,” outlining the emotional aspect of accountability: “…. taking pride in your job and doing it right will show other people that if something goes south your aware of what to do and how things should go.” His comment on work and pride suggests that the accountability presupposes bonds between working bodies and work that are physical and emotional, which suggests an emotional component to correctional work. Such bonds, however, can hinder, and often do so, officers from “being themselves” outside of work and developing work-life balance.

Participants emphasizing attributability occasionally outlined the high level of physical and mental commitment that the correctional work requires from employees and the intensity of this line of work in a person’s life. P112 said, among others, that CO values peers who are “loyal to the job,” as if, to be accountable, people had to pledge allegiance to the job: “Integrity, again for sure, that’s always one of my top ones, being loyal to the job, credible, accountable for things that you’ve done [but] that maybe you shouldn’t [have] done. . . Mistakes. . . Again, trustworthiness is big for me.”

#### Answerability

“Answerability,” the last element we analyzed under accountability, refers to the expectation that employees can reasonably explain and justify their actions according to prison protocols. Answerability appeared in the interviews as doing activities according to rules or “doing everything the correct way.” Some participants also discussed answerability as “not crossing the line.” P20 expected officers to appreciate peers who followed the rules and were not “so willing to cross the line.” When inquired about what he meant by “willing to cross the line,” he said: “ah, like turn a blind eye to something.” Also, using the word “line” as a metaphor for “rule,” P64 said that officers deserving respect from peers are those who obey rules and procedures, doing everything well. According to P64, folks “walking in a straight line” can become the “role model kind of thing.” Meanwhile, others also added an emotional layer to answerability, implying that good officers should embrace a stoic resignation when walking in a straight line: “they follow the rules. They don’t cause drama. I’ve been told that drama can be a hard part about the job….” (P14).

Participants often described “doing the job right” as being diligent. For instance, P2 said: “To try to stay motivated to be diligent about your job and to follow proper procedures.” Similarly, P81 said that “showing respect for everyone else and due diligence as far as your duties go will make peers see you as fit for your position.” P87 framed diligence in terms of caring, as he compared two types of officers, those who complete a task with care and those who do it just for the sake of finishing it.

“If I see a guy and he’s checking for bodies and he’s doing his job properly. . . like every time he goes and does his search, you know he checked for a live body other than a guy that just goes [and] looks and to see if he sees something in the bed.”

P107 added that officers who deserve respect should not only do their job right but also “actually care” about their work: “Conduct yourself as if you give a shit.” In agreement with P107, P52 stated that “great officers” are “just genuinely good, caring people” as opposed to “are more of the self-centered kind of self-serving people so. . .” Also portraying good officers as those who “answer” to the call and needs of others, P47 highlighted the officer’s “willingness to go above and beyond” as a characteristic that he would respect in his peers.

Some recruits discussed answerability in terms of fairness and consistency toward prisoners and following protocols when interacting with the prison population. While P16 said that she “would respect a colleague that …. treated inmates fairly and as if they were a person still.” P60 expected COs to respect peers “who can set the boundaries with the inmates so there’s not you know you have one correctional officer doing this and another doing [that].”

### Reliability

Recruits also described “reliability” as a quality that COs respect in their peers, anticipating the dangers and injury-inducing situations that outline correctional work. Reliability refers to being suitable to rely on. While a few participants (P1, 26, 46, and 63) used the expressions “team” and “teamwork,” most participants said that good officers have “each other’s back.” After considering adherence to rules among other elements that form accountability, P86 said that she wanted to work with someone who shared her values and was going to have her back, stating her personal preference. P150 added reliability to his “wish list” in a peer: “I’d have to say integrity and probably personal safety and making sure you have each other’s backs.” Another participant, P6, used the question topic as an opportunity to discuss the type of officer that he did not want to work with. However, his answer clarified that he too expected that “a good officer would be a team player rather than focused on personal gain. The entitlement to oneself. Just the not wanting to be a part of the team.”

Alluding to the risks of correctional work, P152 and P154 suggested that perceiving peer reliability is a necessity, even a sort of assurance in their line of work: “They need to know that you’re going to be there for them, they need to know that you’re going to back them up, that you’re not going to run away right” (P152); “Because obviously they’ve been through stuff while being there, so to listen to that and to know that you’re going to have their back” (P154). P71, unlike P152 and P154, was more visual in connecting peer reliability to the CO’s safety: “…. Especially with max institutions…. that you know you might be the only two officers out in the yard, and you got tons of inmates on movement …. you know you got to have each other’s backs.” P109 also linked reliability to safety, saying that he would “respect a lot more” officers who helped him in case of an altercation, attack: “…. I’d rather someone kind of join in on it a little bit to help me out…. Come to my aid, don’t just stand there and be like, ‘oh, that’s nice,’ no, like help me.”

Other recruits explored the attributes of reliability in their answers, including trustworthiness, honestly, and loyalty. P100 suggested that reliable officers are trustworthy: “Just being trusting, I mean you’re dependent on, on your partner and other COs if anything goes wrong and if you can’t trust them.” While P26 used the word “genuine” as a synonym for trustworthiness, P106 used “honesty”: “Honesty. I think the ability to trust is the biggest one. You cannot get along with someone and when it comes down to it if you can trust that person that’s the safest and most desirable outcome,” he said associating reliability with occupational safety similarly to other participants. P15 expected good officers to be “helpful” and “honest”: “Helpful. Honest. Not telling you the wrong stuff. Helping you if you need it….” Here, again the emotional elements of correctional work are evidenced in the words of P15, P106 and P100, as trust, helpfulness, and honesty are emotions inherent to safety when working in prison.

Loyalty was another important attribute underpinning the reliability. While P14 expected “loyalty” to be a respected quality among officers, P78 tied loyalty, integrity, and a sense of comradeship into a combo:

“Integrity and I believe, it’s always from what I hear, is loyalty too because it’s like basically, you have to stick, you have to protect one another too, so loyalty for sure because it’s you know, they call it the brotherhood ….”

Recruits who discussed the meanings of reliability more extensively often suggested a preference for officers who try to resolve problems between peers first before escalating in the chain of command. For instance, P12 explained the following:

[having] your officers back. If something needs to be reported, obviously, my personal belief is that you should try to deal with things yourselves. I think you should have your officer’s backs and not run and try to get one up on everyone or try to tell on people. I don’t think that helps in that atmosphere. I don’t think it’s conducive to a safe environment because you want people to have your back too. You have to be very mindful about how and what you do (P12).

Several participants further articulated P12’s point, referring to exposing one’s “wrongdoings” or poor decisions, as well as scrutinizing and judging one’s action as “being a rat.” P62 expected COs to test new recruits to distinguish “rats” from reliable co-workers: “…. they’ll do something that’s not really by policy and then if they get in trouble for it obviously it’s the new recruit that ratted them out, and now, now you’re a rat.” P75 said that “good officers keep their heads down, make sure they have their co-workers’ back, and do not “rat on” their peers, in addition to doing their jobs properly.” Despite not using the “rat” metaphor, P89 also said COs must keep their “nose” to themselves. P89’s and P75’s comments suggest that not interfering in each other’s affairs and not reporting each other are a standard practice among recruits starting in correctional work.

P17 summarized reliability by saying: “Being solid with [your partner] …. covering up your buddy’s ‘crimes’ almost [participant signal air quotes], and …. being there to back them up if something’s happening….Having each other’s back….” However, his summary excluded a commonly discussed facet of reliability: physical fitness; explicitly here was the notion that an unfit officer would struggle to respond to situations, thus, possibly not providing support to their fellow officers. For instance, P90 expected fitness to be a characteristic valued by officers because “people that are not in shape, that can’t respond to a code.” P28 also expected officers to value co-workers who keep themselves in shape, clearly framing fitness as a matter of occupational safety: “Ninety-nine percent of the time nothing happens but it’s the one percent of the time where I need you to help me with a situation or whatever.” Talking about correctional training classmates who allegedly were not physically fit, P122 said:

“[They] would probably die before they would even reach me…. There is a potential safety hazard…. If you can’t even make it to the other side of the basketball court how you are going to be able to fit off an offender.”

Also expecting physical fitness to be the most valued characteristic in a CO, P114 added that he would not want to be “stuck down range” with a classmate that was not fit “if shit hits the fan.” P114 feared getting “freaking beat up you know, like I don’t want that.” Also discussing the physical dimension of correctional work and the safety implications of not being fit, P37 criticized the inexistence of a “physical standard or testing” in the CSC’s recruitment process, calling the situation unfair for those officers who keep fit and always ready to run to help a co-worker:

I just don’t understand like there’s no physical standard or testing; I just think that’s ridiculous. What if I’m on a range you know 300 yards from somebody, and I’m being attacked, and I have to rely on some like one of the girls in our [CTP] class? [Their] hobbies are playing video games all night …. She’s out of shape and I’m just looking at her like I don’t think that because you do this bookwork and stand there and manipulate gun …. that means that you would be a good correctional officer. I just think that’s ridiculous! I think that there should be some serious physical like I don’t feel it’s fair! I train my ass off…. (P37)

### Confidence

Confidence was another recurrent quality that recruits perceived as deserving of respect in correctional work. Participants understood “confidence” as individuals being certain of their abilities. Often, confidence was also described as “assertiveness.” P19, for instance, said that a respected CO should “have that assertiveness and confidence…. It just fits, sends they know you’re confident in what you’re doing.” As P19 further explained his view, he associated the confidence with reliability; he suggested that the COs tend to rely on co-workers who show assertiveness and confidence, using them as a backup: “They’re confident in you, like, being able to protect, not protect them, but have a backup.”

In the eyes of our participants, confidence also had an emotional facet; just mastering working routines was not enough. Recruits expected confident officers to manage their emotions by separating private life, including personal problems, from work life, as P35 put it:

They’re firm and consistent. They know what they have to do. They’re aware of policy and they have good communication and crisis intervention skills. I think just not allowing personal things to become involved in work is a really big part of being a correctional officer because it is a people business altogether (P35).

Discussing emotional management as part and parcel of confidence, P32 said that “officers who stand out do not freeze up; they handle themselves well and think quickly there is a situation are fit for the occupation, demonstrating the value based on managing emotions and the presentation of self.” Similarly, P49 described those officers who have “natural self-confidence” as “not being scared to speak up, not being scared to make a decision without guidance, [being] able to work in a grey area without something being black or white. . .” P126 said that “assertive” officers are not “afraid to ask questions.” In a complementary fashion, P117 portrayed having confidence as being a “calm headed person” who is not going to “raise to that escalation.” P35 associated poor emotion management with increased vulnerabilities in the workplace. According to her, officers who do not manage well with their emotions can be “totally” manipulated by prisoners:

Just quick to emotion, I suppose. Which could make people vulnerable. I don’t want to take the attitude that emotions are weak but that can totally be manipulated by offenders and other officers, but I also think with more life experience certain people could become, you know, more or could be a better CO (P35).

A few participants also associated confidence with respect and care for others. For instance, P123 said that confidence implied “listening to your co-workers taking advice” and not “brushing someone off,” in addition to having “that officer presence.” Meanwhile, a few CORs depicted confidence with attributes that fit traditional standards of masculinities (e.g. stoic and assertive) (Connell, 2005). For instance, although most recruits were attracted to correctional work to “make a change in people’s lives” and showed compassion toward prisoners, some recruits expected COs to respect “100 percent” peers who perform like a “real man”: “Rough, hard-ass, not a con-lover, somebody who probably swears a lot, a real boy…. being a man” (P155).

The tie to masculinities underpins P59 conflating confidence with toughness: “To know that you’re confident in doing something. I guess your toughness?” While P59 tied confidence to toughness, P32 and P61 linked confidence to authority. P32, however, made sure to clarify that being authoritative does not mean being a “hard-ass” who mistreat prisoners: “I feel a lot of us, have the firm but authoritative; not like the hard-ass locking you up and throw away the key. But the ones that are like going in and being fair.” Using the word “assertive” to convey “confidence,” P61 said that a good officer can “give orders and give commands” to other officers, while being “part of the club.” Meanwhile, P120 explored the physical dimension of confidence, saying that people can be confident “even if” they are not “the biggest person.” For P120, being confident is about communication, particularly, the way individuals project their voice. Also emphasizing communication, P131’s views add to P120’s. For P131, good officers do not need to raise their voices to have “a presence”; for her, confidence is an inherent, natural attribute that people either have or do not have. P131 and several other recruits believed that officers who are not confident enough to act quickly and assertively risk being “manipulated” by prisoners. There was a small number of recruits who expected COs to respect peers who follow a standard of masculinities that, today, is widely regarded as an oppressive and unwelcomed. P157, for instance, countered that officers should never exceed to the point of being hated by prisoners. P32 summarized the balance that P155 tried to explain in her comment with the expression “firm but authoritative”; she also associated such a balanced behavior with fairness to prisoners: “…. Firm but authoritative. Not like the hard ass locking you up and throw away the key. But the ones that are [firm but authoritative are] going in and being fair.”

## Discussion

Our findings indicate that recruits perceived good officers as accountable, reliable, and confident. Accountability and confidence express the expectations that underpin correctional work in Canadian federal prisons, while reliability reflects the vulnerabilities that COs face daily because of the dangerous nature of correctional work. CSC socializes CORs to understand both the expectations and the vulnerabilities that mark correctional work during CTP.

The training provided to CORs presents accountability and confidence, although subtly (without necessarily naming those attributes), as necessary and commendable qualities for officers to accomplish the following tasks successfully: make decisions without compromising their legal and institutional safety and take actions, while safeguarding the prisoner’s well-being (CSC, 2018; Ricciardelli, 2021). Emphasized by about half of participants, the value of accountability is transmitted to CORs through the Engagement and Intervention Model (EIM) (CSC, 2018). EIM is the primary framework assisting officer decision-making in case of security/health incidents (CSC, 2018). EIM builds on two principles that embedded in accountability. Expressing answerability, the EIM reinforces officers must justify their actions according to law and policy. Signifying attributability, officers are accountable to their actions. Practically, EIM guides officers on how to “plan” and “execute” their interventions. For instance, when planning interventions, officers must account for the “ability,” “intent,” and “means” relative to a threat, as well as have a clear understanding of the goal motivating their intervention (CSC, 2018). To ensure both attributability and answerability, EIM requires all staff involved in the incident to, among other tasks, submit observation reports and collect evidence pertaining to the incident (e.g. video surveillance records) (CSC, 2018). Correctional training also helps to understand the officers’ emphasis on confidence.

Although the word “confidence” (emphasized by 15 percent of participants) is not mentioned in EIM, the skills that CORs used to exemplify this attribute (e.g. standing out, not freezing up, handling themselves, thinking quickly, and being a good communicator) are indispensable for officers to implement EIM in the heat of the moment. Some of these skills, particularly the ability to “communicate,” which CORs reported as “projecting your voice” when discussing confidence, is presented in EIM as crucial for “cooperation” and “dynamic security” (CSC, 2018). Regardless of the officer’s gender, confidence was usually depicted as a form of masculinities. Depictions of confidence included being assertive, strong (physically), tough, or rough, showing no feelings for prisoners, having emotional control (e.g. “not freezing”), giving commands, being a good communicator (e.g. project the voice), and being authoritative (but fair), that is the standards of behavior that mark traditional masculine socialization processes and identities (Connell, 2005). Conversely, the perception of toughness and roughness as sources of occupational respect and fitness, as well as elements that characterize the CO role (Carter, 1996) can lead recruits to hide their fears, insecurities, and other feelings instead of addressing them. On the flipside, understandings of confidence embedded in traditional masculinities may encourage officers to hide any mental or corporal manifestation that can show their vulnerability against the dangers of correctional work. Suppressing such manifestation can hinder the officer’s ability to deal with the potentially psychologically traumatic events and their outcomes, which often include occupational stress injuries (Carleton et al., 2019; Carleton et al., 2018; Carleton et al., 2020). In a nutshell, the same confidence that empowers officers to “wear” the CO role can trigger feelings of doubt and insecurity.

Our findings regarding accountability and confidence confirm the literature discussing prisons as “emotional places” (Crawley, 2004). While the performative side of accountability (i.e. get the job done) requires, based on interviews, some humor and grace (i.e. using emotions to construct productivity), confidence involves suppressing one’s emotions and the emotions of others (e.g. hostility, aggressiveness, and fear), as well as using emotions to constrain situations and people. Essentially, participants seemed aware that the ability to regulate emotions is an evaluation criterion of performance within correctional work.

Highlighted by over one-third of participants, reliability is another remarkable characteristic defining the “good officer.” CORs expect good fit employees to value “teamwork” and have their “co-worker’s back” while performing the job. Recruits viewed reliability as conducive to occupational safety in the prison environment. Reliability worked as a sort of “life insurance at work” in the case of immediate danger, especially victimization by prisoners (e.g. assaults) (Boyd, 2011). A few CORs viewed physical fitness (i.e. readiness to run or fight to save a co-worker) as signifier of reliability, giving a concrete sense to the idea of “having someone’s back.” Although research done in Australia and the United States (Liebling et al., 2011) has shown that male officers sometimes have concerns about counting on female officers as a physical backup, that was not the case among our study’s participants; only the co-worker’s ability and willingness to physically react to an incident mattered.

A few recruits exemplified reliability as not reporting the “wrongdoings” of co-workers (i.e. “rat someone out”). Noteworthy, participants have used the expression “wrongdoings” in reference to controversial decisions (e.g. “not really according to policy”), which, based on our research experience and the reports of others (Liebling et al., 2011), also include bending the rules to favor prisoners, as opposed to actual serious violations and crimes such as officer-on-prisoner assault and violence against prisoners. Not “ratting out” co-workers, depending on the case, may contradict accountability (including attributability and answerability) and raise ethical concerns that can and are addressed through training. However, these “spoiled” versions of reliability do not derive from the officer’s compromised morality or identity. Instead, this expectation is rooted in structural factors, particularly the need to stay safe in an environment that is marked as dangers and risks, as well as organizational problems (e.g. short staffing and high turnover rates) (Public Services Foundation of Canada, 2015), which make officers significantly and excessively dependent on their peers. Overall, our findings on reliability demonstrate that the ability to rely on co-workers is more than a form of social interaction in the workplace. It is a tool correctional that officers utilize to manage the occupational risks and “occupational stress injuries” that mark their life at work (Carleton et al., 2019; Carleton et al., 2018; Carleton et al., 2020; Oliphant, 2016;, Ricciardelli2019b). Reliability also seems to be a core feature of correctional work subcultures, which researchers focused on prison employee culture have left unexplored (Arnold et al., 2007; Duffee, 1974; Farkas and Manning, 1997; Ingel, 2020; Klofas and Toch, 1982) and thus a potentially significant site of further investigation than can impact stress (Lambert et al., 2006) and job satisfaction within correctional work.

Accountability, confidence, and reliability creates a regulatory environment that helps in the governance of the CO role. Confidence and reliability meant analyzing work-related situations, making decisions, and taking action that can be reasonably justified because they comply with rules and protocols. In other words, accountability and confidence give officers agency within correctional work, preparing CORs to “wear” their occupational role; a role that includes dealing with the continual scrutiny of policies, protocols, and the gaze of others, including colleagues, managers, prisoners, and society (through the publicity that some events receive), as well as bear the consequences of their actions. Meanwhile, reliability exposes their occupational vulnerability not only as workers who want to survive their job and go back home safe, (Ricciardelli2019a, 2019b) but also as individuals who have to stand by their actions when under scrutiny/or investigation.

The regulatory environment of which accountability, confidence, and reliability are a part contribute to a moral code of conduct that helps to govern what prison researchers in the UK have called “moral performance” (Liebling and Arnold, 2004). Moral performance refers to how prisoners and staff “see” and “feel” they are morally treated in prison (Liebling and Arnold, 2004). Practically, our findings suggest the officers (as well as prisoners) view, feel, and experience accountability, confidence, and reliability may reveal the quality of prison as a place of incarceration and work, as well as job satisfaction. The themes emerging in our analysis can contribute to the literature operationalizing job role and its impact on job satisfaction and stress (Cullen et al., 1985; Dowden and Tellier, 2004; Lambert et al., 2006; Lambert et al., 2002; Whitehead and Lindquist, 1986). Our findings on this regulatory environment marked by accountability, confidence, and reliability also signal that correctional services are up-to-date with broader societal trends involving organizational governance, particularly employee agency and independent problem-solving capacity (Child and Rodrigues, 2003).

We found no association between participants’ views of occupational fitness and their gender, age, and correctional experience. This finding seems to be in line with previous research on the topic of role and occupational fitness, which also did not identify macro-level differences in how COs of different genders perform their role (Arnold et al., 2007; Crawley, 2004; Liebling et al., 2011). However, efforts to identify such factors should still be pursued, as they might certainly exist when analyzing the CO expectation involving micro aspects of the work routine. Identifying the determinants of co-worker expectations and occupational fitness will help correctional services further improve employee expectations’ governance and thus improve CO’s overall well-being.

## Conclusion

A window into the recruit’s occupational mind-set, our findings support that prison officers are not “omnipotent rulers who have crushed all signs of rebellion against their regime” (Liebling et al., 2011; Sykes, 1958). Officers are prepared to use force in correctional institutions, however, force use shall be legitimate, that is, embedded in accountability, confidence, and reliability, qualities that structure the CO role and regulate social relationships among officers, as well as between officers and prisoners. Our findings also suggest that COs, like police officers (Van Maanen, 1975), start to take on the CO role, as well as participate in the subcultures that characterize correctional work (Arnold et al., 2007; Morrison and Maycock, 2021), through a process of “organizational socialization” that starts in training and recruitment. Although consistent and controlled, socialization accommodates diversity and flexibility in how officers perform the CO role; the variation seen in the participants’ views and interpretation of the qualities outlining the role model officer, as well as the tensions embedded in the data (e.g. loyalty is valued among colleagues, but not necessary to prisoners or management), signify this diversity, as well as the complexity of the CO role (Liebling et al., 2011).

Our study includes limitations related to its design and generalizability. From the design perspective, our analysis excludes occupational fitness factors associated with the management of prisoners. Although the broader study facilitating our analysis inquiries into the CO’s perceptions of what prisoners would consider a good officer, we decided to discuss occupational fitness from the perspective of the COR and the prisoner separately to preserve the analytical depth and facilitate future comparisons of the two perspectives. From an international perspective, our study includes characteristics that limit generalizability (e.g. a federal Canadian sample and, like any concept, the meanings of accountability, reliability, and confidence vary culturally).
